# Experiment and analysis on the heterogeneity of CO_2_ adsorption-transport by microrocks in coal matrix

**DOI:** 10.1371/journal.pone.0314162

**Published:** 2025-03-06

**Authors:** Guosong Jing, Qian Zhao, Ziqiang Jin

**Affiliations:** 1 School of Transportation Engineering, Huanghe Jiaotong University, Henan, Jiaozuo, China; 2 Department of Civil Engineering, Henan Polytechnic University, Henan, China; Shenyang Jianzhu University, CHINA

## Abstract

In order to study gas adsorption performance effected by micro-rock in coal matrix, dual energy X-ray CT was used to calculate the density change of coal core before and after gas injection, which can obtain the CO_2_ concentration (adsorption amount) in coal core, and reveal the uneven characteristics of CO_2_ adsorption by minerals. The research results show that coal core in the region where minerals exist has a higher density, while the density of the coal matrix is smaller. Regions with higher coal density (more minerals) have weaker adsorption capacity for CO_2_. The CO_2_ concentration in coal core decreases approximately linearly along the axial direction of coal core from the gas injection end to the outlet section. The average voxel density is basically the same at the same coal core section before and after gas injection, which indicates that the coal matrix recovers and approaches the original state after desorption. However, before and after gas injection, the average voxel density of coal core varies greatly, and the frequency variation and average deviation factor of different voxel densities vary greatly, which indicates that the adsorption of CO_2_ by coal core is extremely uneven.

## 1 Introduction

In the process of CO_2_ enhanced coalbed methane (ECBM) production/storage, injecting CO_2_ into coal seams will not only increase the pressure gradient to drive methane flow, but also replace the methane adsorbed in the coal matrix by reducing the partial pressure of methane in the gas mixture [1,2]. The adsorption capacity of coal for gases can not only be used to predict the maximum possible volume of gas stored in coal, but also to predict the amount of gas released from coal during production. Numerous scholars have studied the gas adsorption capacity of coal using coal powder [3,4]. Although these studies provide a relatively fast method for estimating coal storage capacity, in practical situations, coal is not only in a three-dimensional stress state, but also does not exist in powder form [5]. Moreover, a large number of experiments have shown that coal has a much greater adsorption capacity for CO_2_ than methane, and coal seams have enormous potential for sequestering carbon dioxide [6,7]. Therefore, it is necessary to study the adsorption capacity and diffusion effect of CO_2_ in coal matrix under stress, which is also an important parameter for the technical and economic indicators of CO_2_ storage in coal reservoirs [8].

Research has shown that coal contains a large amount of minerals, and the gas permeation diffusion process in the coal matrix also exhibits anisotropic characteristics, while gas adsorption also exhibits non-uniformity [9–11]. The mineral content in coal ranges from 10% to 25%, with some low rank coal having a mineral content of up to 40% [12,13]. The mineral composition in coal is relatively complex, mainly consisting of vitrinite, inertinite, and chitin. The distribution of minerals in coal not only affects the pore structure, but also affects the gas adsorption ability of coal [14–16]. For the ability of minerals to adsorb and store gas in coal, most scholars believe that the Langmuir volume of the same coal quality decreases with the increase of ash content. The pore characteristics of coal are analyzed, minerals fill a certain proportion of micropores in coal, thus reducing the specific surface region of coal, and thus reducing the adsorption amount of gas. At the same time, minerals fill the cracks in the coal matrix, thus reducing the permeability of gas in the coal seam [17–20].

Regarding the adsorption mechanism of coal on gases, some scholars have used X-ray scanning to observe the transportation and storage of gases in the coal core during CO_2_ injection, and analyzed the adsorption rates of different coal on gases through CT image processing [21]. Smith [22] measured the adsorption capacity and diffusion rate of unconfined coal cores using environmental pressure gravity technology; The results indicate that CT can not only provide qualitative measurements of spatial changes in CO_2_ concentration within the core, but also provide quantitative and accurate measurements of CO_2_ adsorption. Liu et al. [23] quantitatively analyzed the damage of power ultrasound stimulation on multi-scale pores and cracks in coal based on gas adsorption and CT image 3D reconstruction. Li et al [24] quantitatively characterized the physical properties of coal using non-destructive low field nuclear magnetic resonance (NMR) and X-ray computed tomography (X-CT). The results indicate that with the increase of stress, the volume of mesopores, macropores, and cracks sharply decreases. Zhou et al. [25] used the method of SEM-EDS and micro CT scanning to observe the microstructure deformation of middling coal during methane adsorption desorption cycle. The results indicate that the deformation of coal during methane adsorption is mainly caused by the expansion and compression stress between different regions. The deformation of clay minerals is mainly influenced by the deformation of their adjacent coal matrix, and residual deformation in coal mainly exists in areas with strong density non-uniformity. Zhou et al. [26] conducted extensive research on the deformation of the microstructure during coal adsorption of methane and found the localized density homogenization effect of the microstructure during coal adsorption of methane. By establishing a new coal structure model, the mechanism of local density homogenization effect was analyzed, and the local density homogenization equation for ethane adsorption in coal was derived. Yu et al. [27] used digital image subtraction technology to extract the structural characteristics of pores and fractures and fluid distribution. Explore the fluid ascent behavior in a single capillary and the flow characteristics within the pore fracture network, in order to reveal the mechanism by which pore fractures affect fluid migration during self suction. Although these studies analyzed gas adsorption, deformation, and damage in coal, they did not investigate the non-uniformity of gas adsorption and transport process.

In order to study the influence of micro rocks (minerals) in coal on its diffusion adsorption, dual energy X-ray CT was used to measure the gas transport and storage in coal core during the CO_2_ injection process, so as to obtain the temporal variation of CO_2_ concentration in coal core. Moreover, the density variation characteristics of different regions affected by minerals and the non-uniformity of CO_2_ adsorption transport were compared and analyzed. In sections 2, 3, and 4, the experimental equipment, methods, and experimental processes are described. The experimental analysis content is in Section 5: (1) Analysis of density differences in coal-body regions; (2) Analysis of liquid nitrogen adsorption and pore structure differences in coal-body regions; (3) The spatiotemporal variation characteristics of coal core gas adsorption under different injection pressures; (4) Characteristics of uneven changes in gas adsorption-desorption of coal core; (5) Heterogeneous changes in gas adsorption-desorption of coal core.

## 2 Coal samples and experimental system

**Fig 1:**
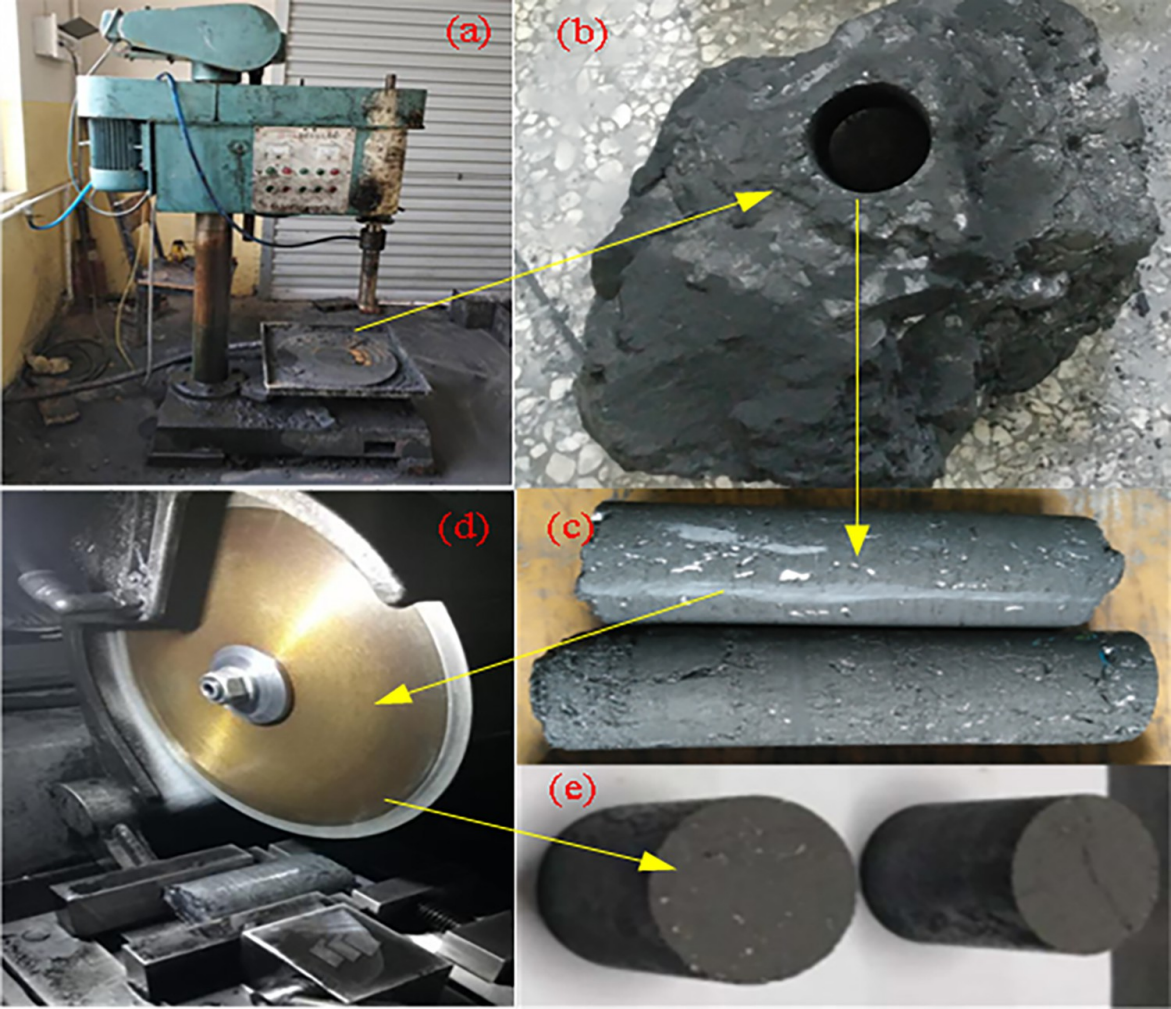
Fabrication of coal specimens.

The experimental coal sample was taken from the raw coal block of the No. 21 coal seam in Jiulishan Coal Mine, Jiaozuo. The HZ-15 electric corer (Fig 1A) was used to core the raw coal block (Fig 1B) with a diameter of 25mm (Fig 1C), and the coal pillar was cut and polished using a TCHR-II cutting mill (Fig 1D) to produce a coal core with a length of 50mm, as shown in Fig 1E.

Put three coal samples into a drying oven, set the temperature to 40°C, dry for 3 days, and then put them into a closed bag for backup. The weight losses of the three coal samples are 0.55%, 0.52%, and 0.57%, respectively. This indicates that during the drying process at 40°C, only the external moisture of the coal sample is removed, and its inherent moisture is difficult to remove.

**Fig 2:**
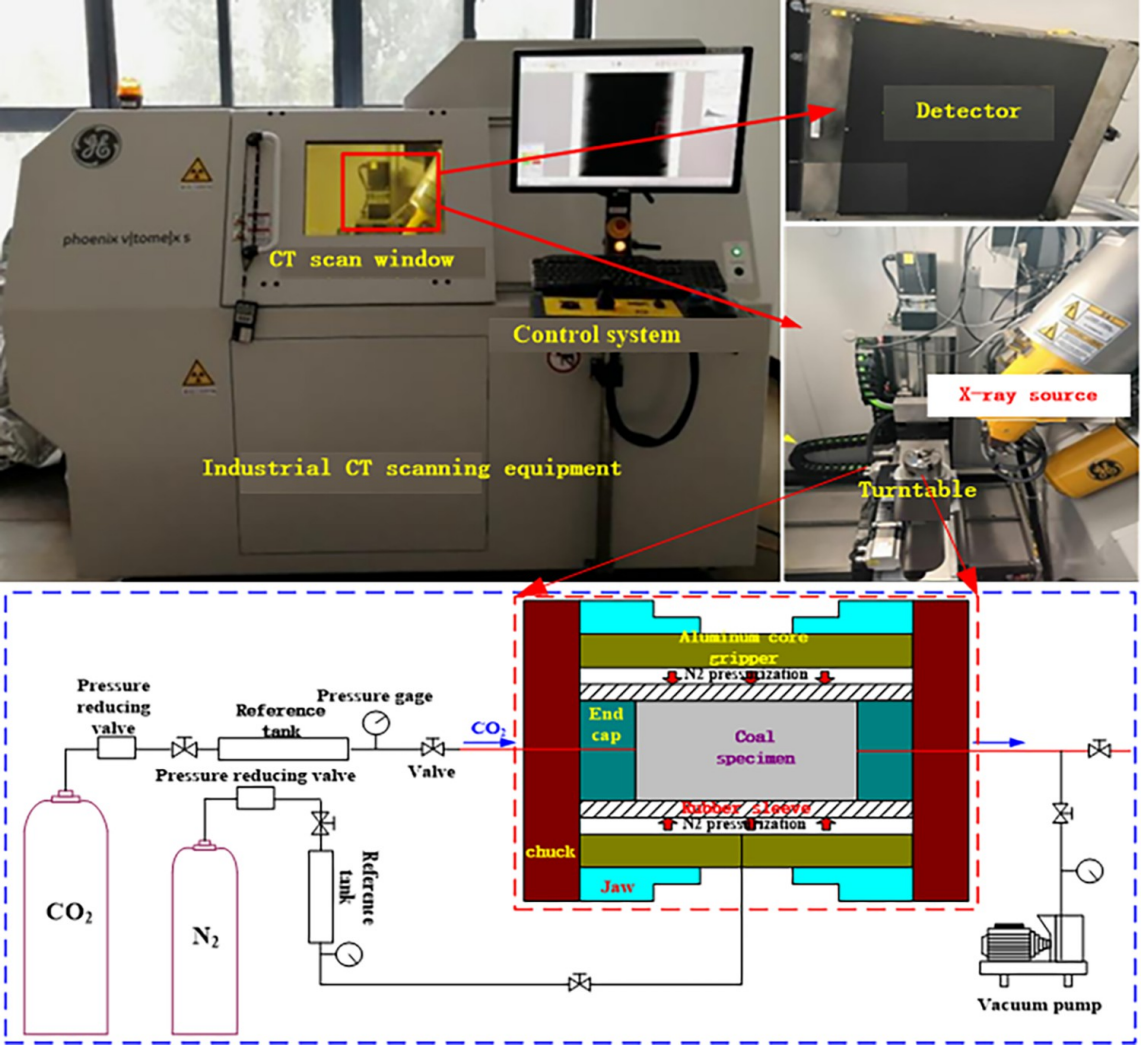
High precision CT scanning instrument and coal sample gas injection system.

The Phoenix v|tom|x s industrial CT scanning system introduced from the key laboratory of Henan Polytechnic University is adopted, as shown in Fig 2. The industrial CT scanning system mainly consists of X-ray source, turntable, coal sample gas injection, coal sample clamping system, X-ray detector, and computer data processing unit.

## 3 Testing methods

When X-ray passes through an object, it will produce complex physical processes such as Photoelectric effect and Compton effect. Part of the X-ray is reflected, scattered and absorbed by the substance, and the intensity of the X-ray is attenuated [28]. The linear attenuation coefficient of a material corresponds to the density of the material. The linear attenuation coefficient of a sample composed of various substances is equal to the sum of the product of the linear attenuation coefficient of the different components and the mass percentage.

Due to the uneven composition of coal, the attenuation of X-rays varies after passing through different regions, and some photons cannot reach the flat panel detector. Some photons can reach the detector through an object and be visualized by a computer controlled system. If the CT value is converted into a certain proportion of grayscale values, the corresponding CT image can be obtained. The bright colors in the CT image represent the high-density regions of the coal body, while the dark colors represent the low-density regions.

When scanning a cylindrical coal core, a digital image is produced. These images reflect the value of the attenuation coefficient in the axial plane (i.e., the “slice”) of the object of study. Attenuation depends on electron density, but can usually be calibrated by conventional weight density. If only one energy level is used, the only quantitative information obtained is the local value of the linear attenuation coefficient, which may not be sufficient to analyze the interface density of the “slice” in detail [29]. Therefore, dual energy scanning technology was used in this experiment [30].

The density of the scanning material is a linear function of the low energy [CT_low_] and high energy [CT_high_] CT values, and its relationship can be expressed as [31].


$$\rho =a[CT_{high}]+b[CT_{low}]+c$$ (1)

Here, the CT values of three reference materials (Table 1) are used to determine the coefficients *a*, *b*, and *c* in the CT value calculation equation. Furthermore, using these coefficients, the density of the coal core under vacuum and the density of the coal core during CO_2_ adsorption can be calculated. When the density values at each time and pressure are obtained, the volume and porosity of the coal sample can be obtained, and thus the CO_2_ adsorption amount can be calculated.

**Table 1:** CT Values of reference materials.

Reference materials	CT value	
	130kV	80kV
Water	-95	11.5
Silica	1162	1755
Aluminium	1780	2725

Based on calibration measurements (see Table 1) and Eq 1, three Equations for water, silica, and aluminum can be obtained:

$$\{\begin{array}{l} 1.000g/{\mathrm{cm}}^{3}=a[-95\mathrm{CTN}]+b[11.5\mathrm{CTN}]+c\\ 2.200g/{\mathrm{cm}}^{3}=a[1162\mathrm{CTN}]+b[1755\mathrm{CTN}]+c\\ 2.699g/{\mathrm{cm}}^{3}=a[1780\mathrm{CTN}]+b[2725\mathrm{CTN}]+c \end{array}$$ (2)

According to Eq (1) and the CT values measured at 80 kV and 130 kV, the calculated values of parameters *a*, *b*, and *c* for mass density are: *a* = 2.71 g/cm^3^, *b* = -0.81 g/cm^3^, and *c* = 1206.23 g/cm^3^, respectively.

## 4 Testing process

**Fig 3:**
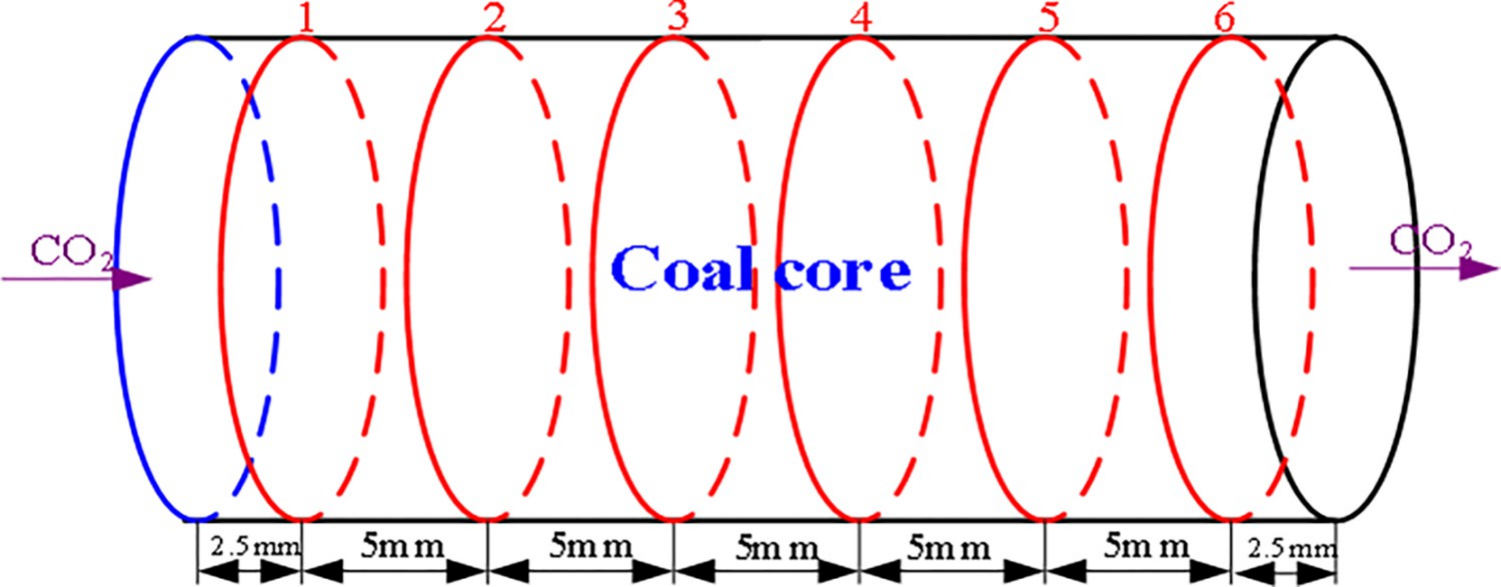
Scanning slice location of coal core.

The rock gripper can apply radial confining pressure (N_2_ pressurization) along the coal core and inject CO_2_ at one end. In this experiment, CO_2_ pressure is applied, and the other end is connected with the atmosphere, so that the CO_2_ adsorption kinetics can be observed at different positions (section slices) along the whole length of the coal core. The test steps are as follows (room temperature):

1. The assembled core holder is installed on the test turntable in the industrial CT scanning system. The coal core is scanned into 2mm thick slices at different locations, and each measured CT number represented 0.25×0.25×2.0mm three-dimensional pixels. 6 scans are carried out along the axial direction (Fig 3).
2. Confining pressure is provided by injecting N_2_ via pressure regulator. During each pressure period of the experiment, the pressure remains constant.
3. Use a vacuum pump to vacuum the coal core until the internal pressure is below 20Pa.
4. Under an effective confining pressure of 1.5MPa, the coal core was scanned and vacuumed at two energy levels of 130 kV and 80 kV.
5. CO_2_ is injected into one end of the coal core. Due to the small size of the coal core, the volume of CO_2_ in the cylinder is relatively large. Therefore, constant CO_2_ pressure is also maintained at the injection inlet. In the gas injection test, the pressure difference between the confining pressure and the inlet CO_2_ pressure remain constant at 1.5MPa.
6. At CO_2_ injection pressures of 1.0 MPa (confining pressure of 2.5 MPa), 2.0 MPa (confining pressure of 3.5 MPa), 3.0 MPa (confining pressure of 4.5 MPa), and 4.0 MPa (confining pressure of 5.5 MPa), the coal core at each section position is repeatedly scanned at the set CO_2_ permeation and adsorption time, and the continuous injection time is 150h.
7. After CO_2_ injection (pressure is 4.0 MPa) is completed, the coal core is desorbed for 50 hours, and the coal core is repeatedly scanned at each slice position.
8. The average density of each section at each time is calculated from its average CT value (Eq 1) and the *a*, *b*, and *c* values. After CO_2_ injection, the density of each slice is subtracted from the average density of the corresponding slice under vacuum, which is the density increment of the slice caused by CO_2_ adsorption. The average CO_2_ density of these slices (unit: mass of CO_2_ per unit volume of slice).
9. The analysis is performed using the VoxelCalc NDT and Image J software processing packages, and color images are generated to illustrate the spatial distribution of density in each slice before or after adsorption occurred.

## 5 Test results and analysis

### 5.1 Coal core density after vacuum extraction

**Fig 4:**
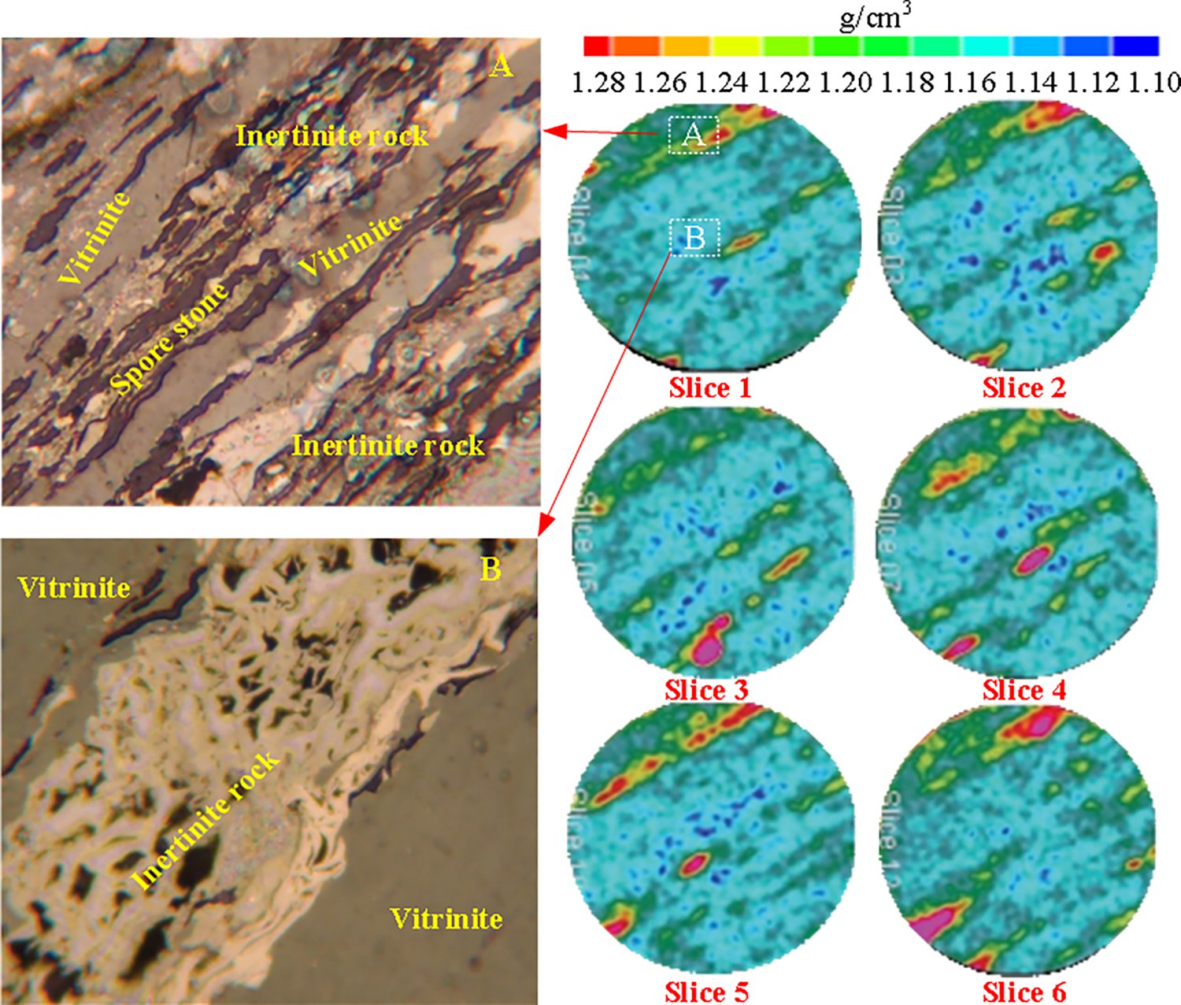
Scanning density change of vacuum coal core.

**Fig 5:**
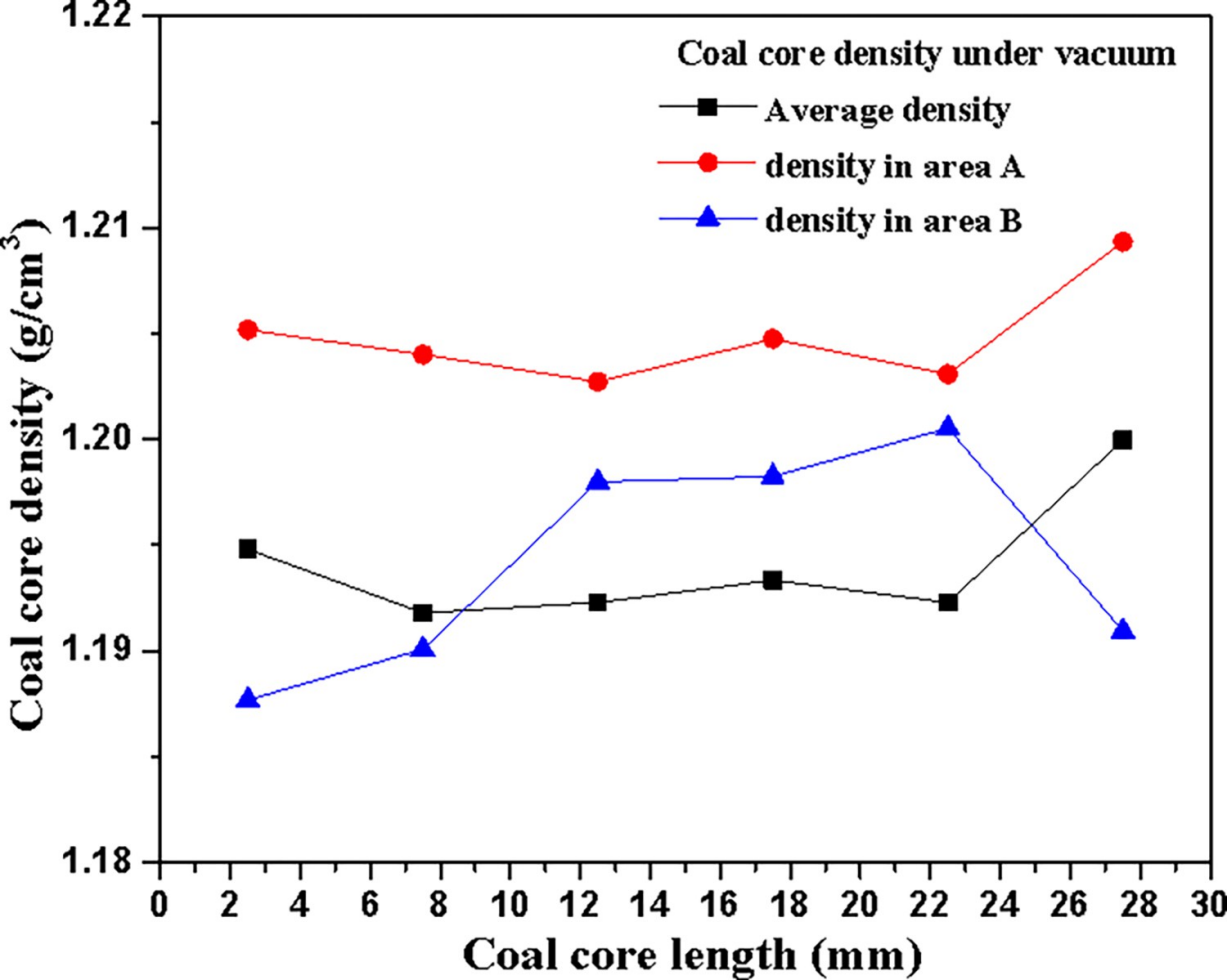
Minerals in high density region of coal core.

After vacuuming the coal core, the density changes of each cross-section of the coal core obtained through scanning calculation are shown in Fig 4. Baby blue indicates that the density of coal matrix is small, while red indicates that the coal contains minerals with high density, as shown in Fig 5 (regions A and B in Fig 4). From Fig 4, it can be seen that the upper left and middle regions of each slice exhibit a higher density of coal specimens (influenced by minerals). The macerals in these regions are closely mixed and mainly composed of three components (vitrinite, sporolite and inertinite). In Fig 4A, sporolite and inertinite dominate the region as consistent bands, and vitrinite zone alternates with sporolite and inertinite mixed zone; The vitrinite in Fig 4B is mixed with meshed inertinite. The average bulk density of coal specimens calculated based on CT data is *ρ* = 1.194 g/cm^3^, and the density of coal samples calculated based on mass and volume is 1.21g/cm^3^, with an error less than 1.40%.

As can be seen from Fig 5, the core is not uniform in each slice and along the core axis. In order to compare and analyze the non-uniformity on the coal core slice, two regions A and B are selected on the coal core slice (as shown in Fig 4). The density of each slice of coal core in these two regions (before vacuuming and gas injection) is calculated according to the CT values of regions A and B. Fig 5 shows the axial change curve of the average density of regions A and B and different slices along the coal core. There is a big difference between the coal density and the average slice density in region A and B. The coal core density in region A is higher than the average slice core density, but its change along the axial direction of the coal core tends to the change rule of the average density. The core density in region B is always lower than that in region A, and it fluctuates greatly along the core axis.

**Fig 6:**
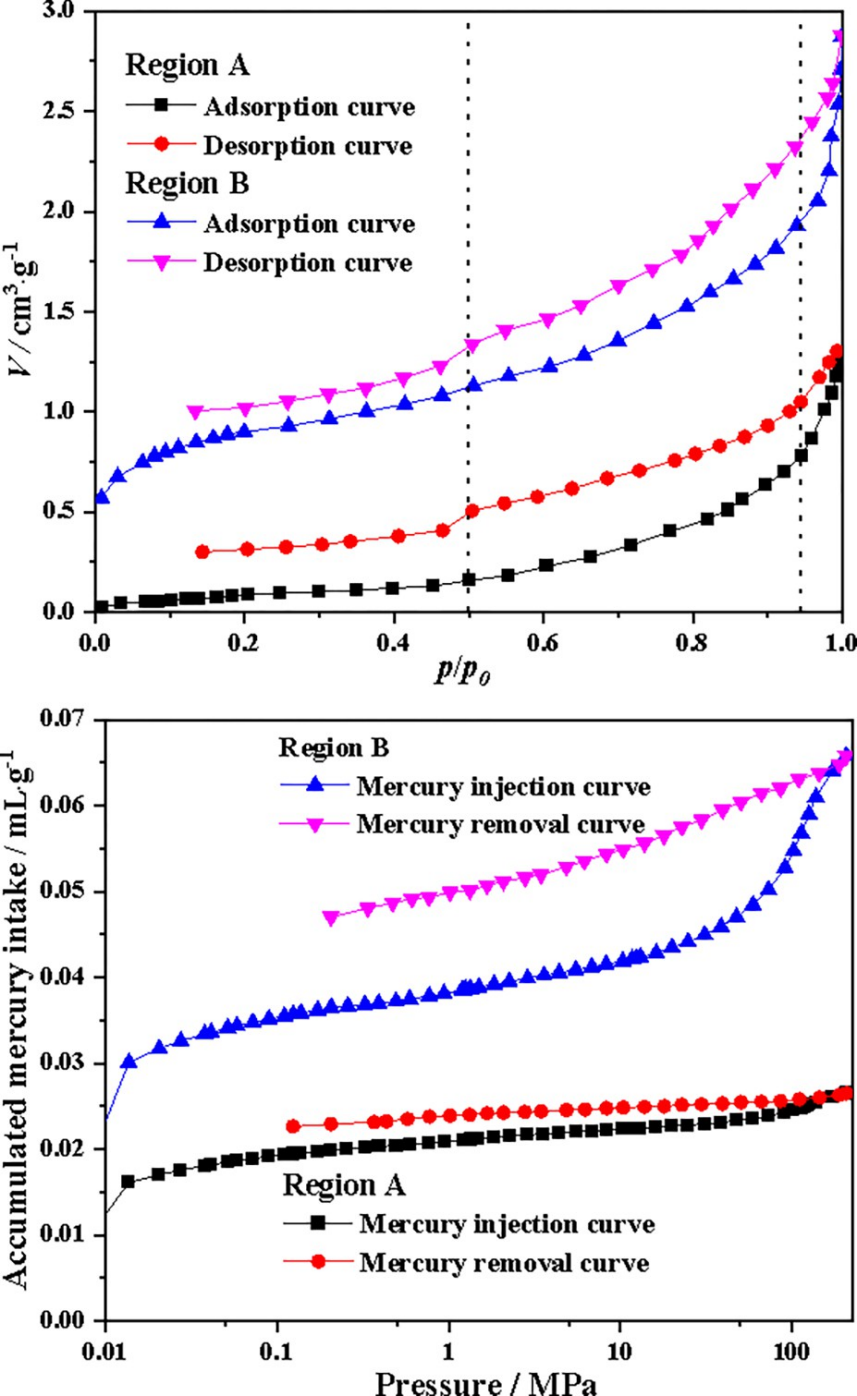
Liquid nitrogen and mercury intrusion test curves of coal samples (a) Liquid nitrogen test curve (b) Mercury intrusion test curve.

Coal samples are collected from Zone A and Zone B of Slice 1 for liquid nitrogen adsorption and mercury intrusion tests to obtain their pore structure characteristics. The liquid nitrogen adsorption/desorption curves and mercury intrusion/desorption curves in coal samples from regions A and B are shown in Fig 6. From Fig 6A, it can be seen that the adsorption and desorption curves exhibit an inverted S-shape, belonging to the type II adsorption isotherm. When the relative pressure is less than 0.5 (*p*/*p*_0_<0.5), the adsorption capacity slowly increases, the adsorption isotherm protrudes upwards, and the first adsorption layer roughly disappears. As the relative pressure increases, the second adsorption layer begins to form on the first adsorption layer. The relative pressure (*p*/*p*_0_) increases from 0.5 to 0.95, and the adsorption curve sharply rises until the vapor pressure approaches saturation (1>*p*/*p*_0_>0.95) before the adsorption saturation phenomenon occurs. For the adsorption of low-temperature liquid nitrogen in coal, single-layer adsorption occurs under surface tension under low pressure (*p*/*p*_0_<0.5); Under medium pressure (0.5<*p*/*p*_0_<0.95), multi-layer adsorption occurs under the action of van der Waals forces; Under high pressure (*p*/*p*_0_>0.95), capillary condensation occurs. The mercury intrusion and mercury removal curves of coal samples in regions A and B are shown in Fig 6B. The mercury injection curve is S-shaped, with a convex curve at low pressure and a concave curve at high pressure. The cumulative amount of mercury entering the coal samples in Region A and Region B gradually increases. Compared with the mercury intrusion hysteresis loop of Region A coal sample, the mercury intrusion hysteresis loop of Region B coal sample is wider, and the pores are mainly open pores.

**Fig 7:**
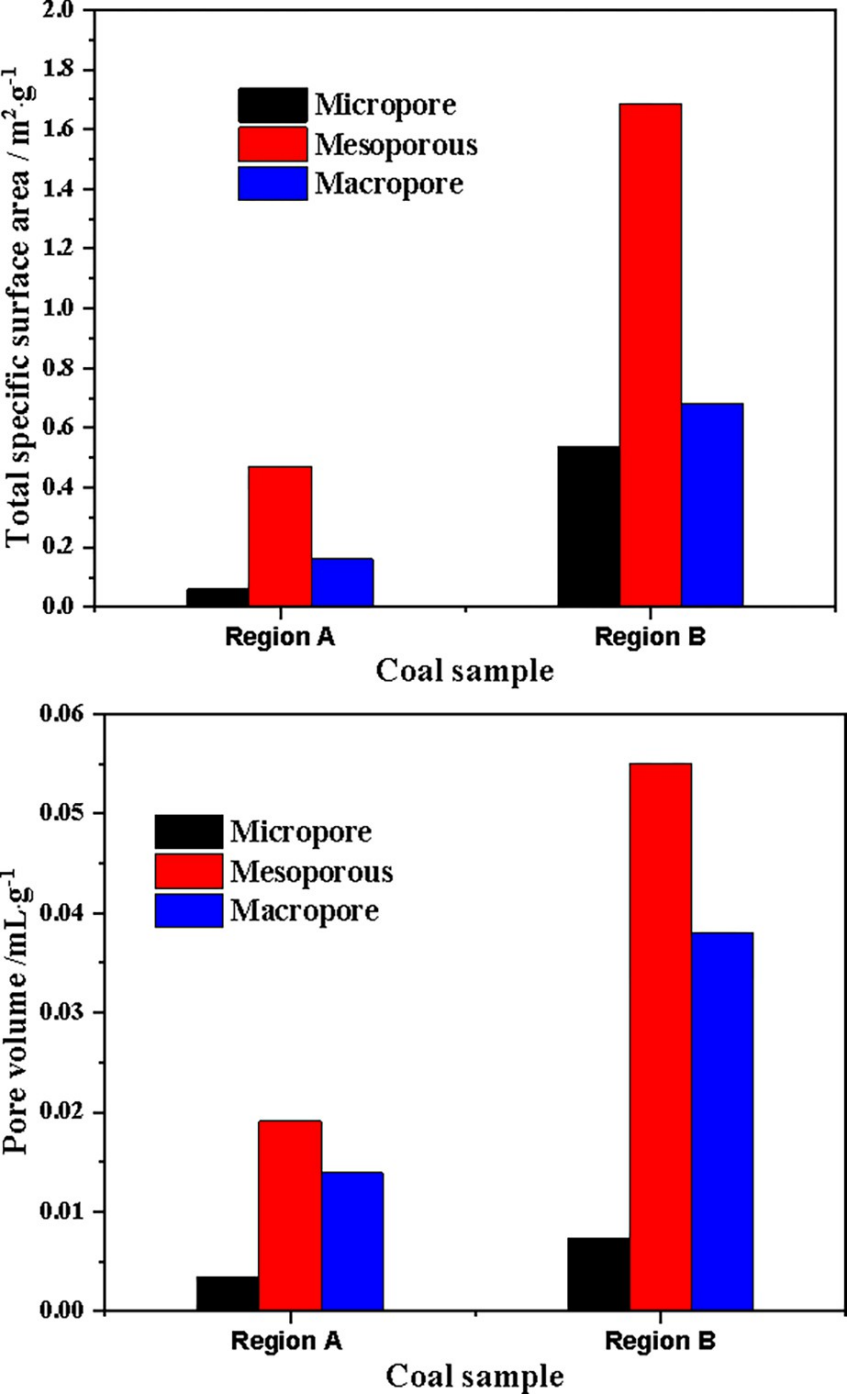
Specific surface area and pore volume of coal samples (a) Specific surface area (b) Pore volume.

According to liquid nitrogen adsorption/desorption curves, the specific surface areas of micropores, mesopores, and macropores in coal sample are shown in Fig 7A. The specific surface areas of micropores, mesopores, and macropores in region A coal sample are 0.0605m^2^/g, 0.4725m^2^/g, and 0.1611m^2^/g, respectively, with a total specific surface area of 0.6941m^2^/g, which is smaller than the specific surface areas of micropores, mesopores, and macropores in region B coal sample of 0.5354m^2^/g, 1.687m^2^/g, and 0.6833m^2^/g, and a total specific surface area of 2.9057m^2^/g. According to the mercury intrusion/mercury removal curves, the pore volumes of micropores, mesopores, and macropores in coal sample are shown in Fig 7B. The pore volumes of micropores, mesopores, and macropores in region A coal sample are 0.0035cm^3^/g, 0.0191cm^3^/g, and 0.0139cm^3^/g, respectively, with a total pore volume of 0.0365cm^3^/g, which is smaller than the pore volumes of micropores, mesopores, and macropores in region B coal sample of 0.0074cm^3^/g, 0.055cm^3^/g, and 0.038cm^3^/g, and a total pore volume of 1.004cm^3^/g. This is mainly due to the high content of minerals (such as inertinite and spore stones) in Region A, while the mineral content in Region B is relatively low. Therefore, the specific surface area and pore volume of Region A coal sample are smaller than those of Region B coal sample, which will lead to differences in the adsorption capacity of Region A and Region B coal samples.

### 5.2 CO_2_ adsorption-migration characteristics under different injection pressures

In this experiment, CO_2_ in the reference tank and auxiliary pipeline (Fig 2) is a high-pressure gas source at the end face of coal core, which forms gas pressure difference with the inside of coal body. Under the pressure difference, the gas penetrates into coal body, which is similar to the coal permeability test [32]. This process is different from the instantaneous release of high-pressure CO_2_ to fracture coal body, which is an extremely slow process [33]. Therefore, the damage effect of high-pressure gas source on coal body can be ignored.

**Fig 8:**
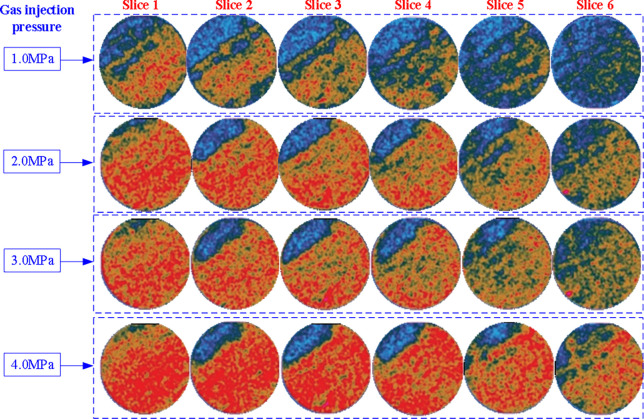
Change of CO_2_ density in coal core at different injection pressures.

CO_2_ was injected at 1.0 MPa (first injection pressure, confining pressure of 2.5 MPa), 2.0 MPa (second injection pressure, confining pressure of 3.5 MPa), 3.0 MPa (third injection pressure, confining pressure of 4.5 MPa), and 4.0 MPa (fourth injection pressure, confining pressure of 5.5 MPa). After 150 hours of injection, the spatial distribution of CO_2_ density in coal core after CO_2_ adsorption (average profile value) is shown in Fig 8 (scan positions 1–6 in Fig 3).

**Fig 9:**
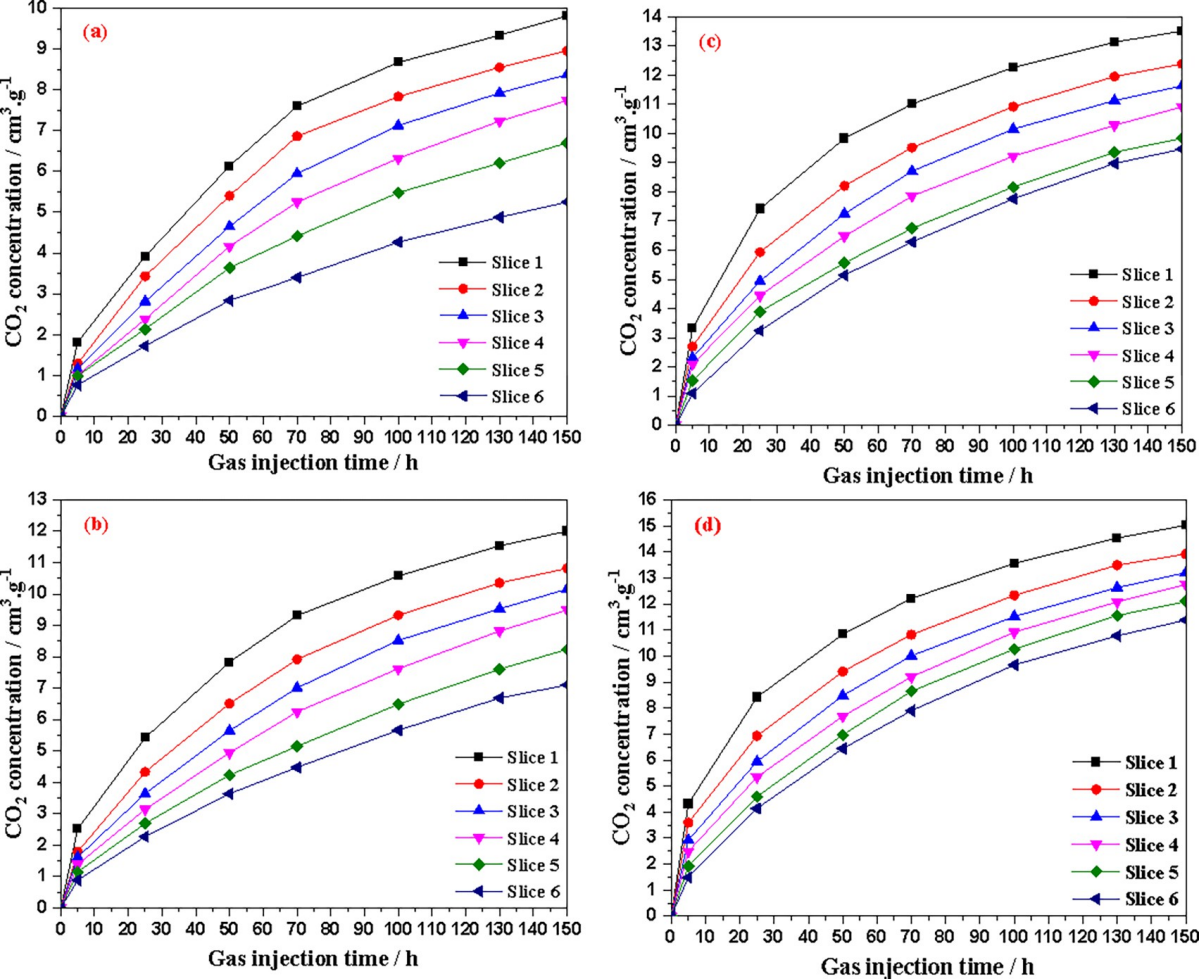
Relationship between CO_2_ concentration and injection time in different slices under different pressures (a) 1.0MPa (b) 2.0MPa (c) 3.0MPa (d) 4.0MPa.

As shown in Fig 8, the coal core slices farther away from the injection inlet contain less CO_2_. In addition, it can be seen that the CO_2_ adsorbed at the coal core slices is uneven, and the upper left part of each slice absorbs less CO_2_. This is because the upper left part of coal core has more minerals and weaker CO_2_ adsorption capacity. Meanwhile, it can also be seen from Fig 9 that the higher the CO_2_ injection pressure, the higher the CO_2_ density at the same position in coal core after the injection is completed (150 hours). That is to say, the higher the CO_2_ injection pressure, not only increases the adsorption capacity of coal for CO_2_, but also promotes the migration of CO_2_ in coal core (from the inlet end to the outlet end). From Fig 9, it can also be seen that the CO_2_ infiltration-diffusion-adsorption process in coal core has not reached equilibrium within 150 hours. Moreover, the further away from the gas injection end, the smaller the amount of CO_2_ adsorbed, that is, the smaller the volume of CO_2_ adsorbed per unit mass of coal core.

**Fig 10:**
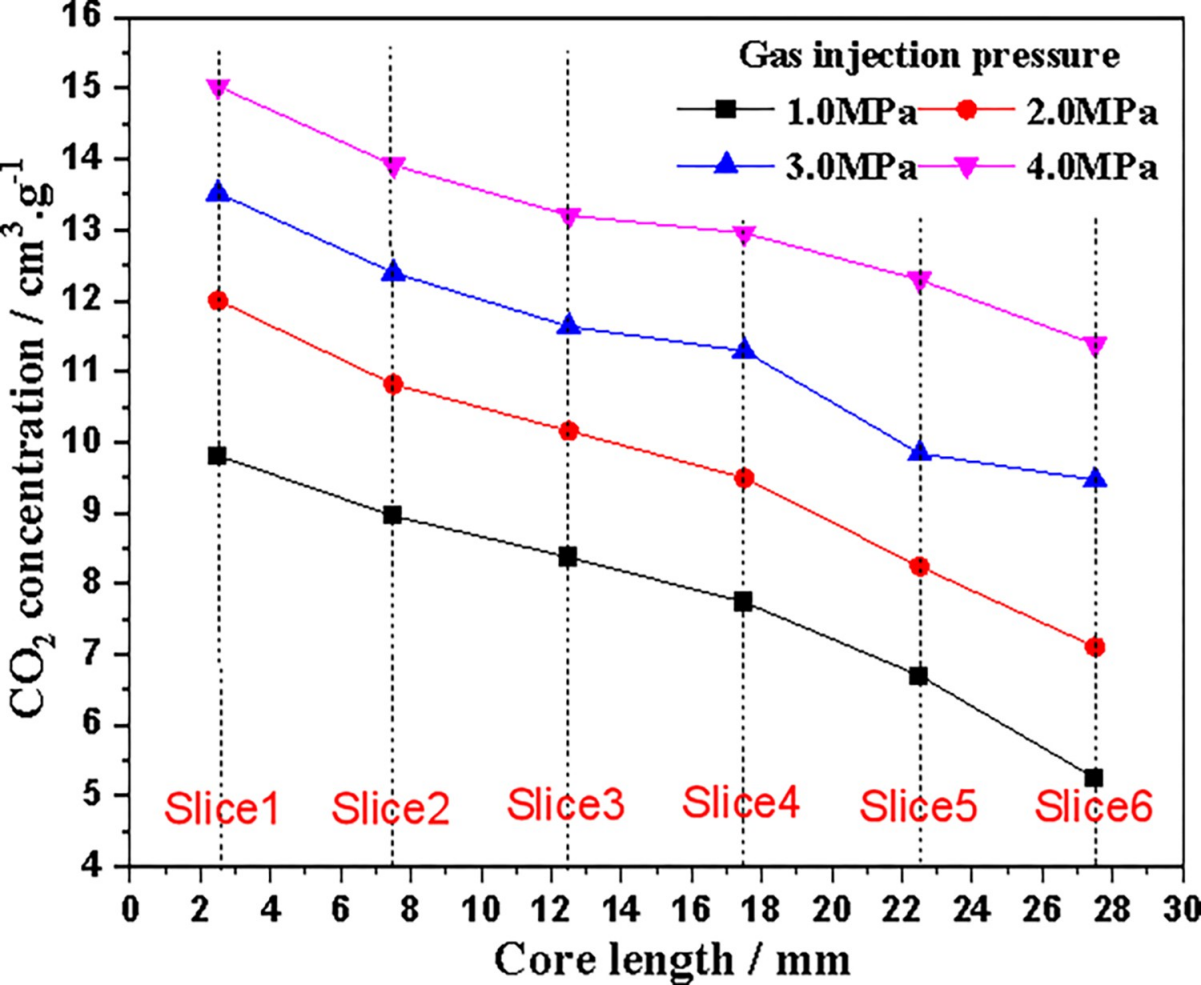
Changes of CO_2_ concentration along the axial direction of coal core after gas injection.

**Fig 11:**
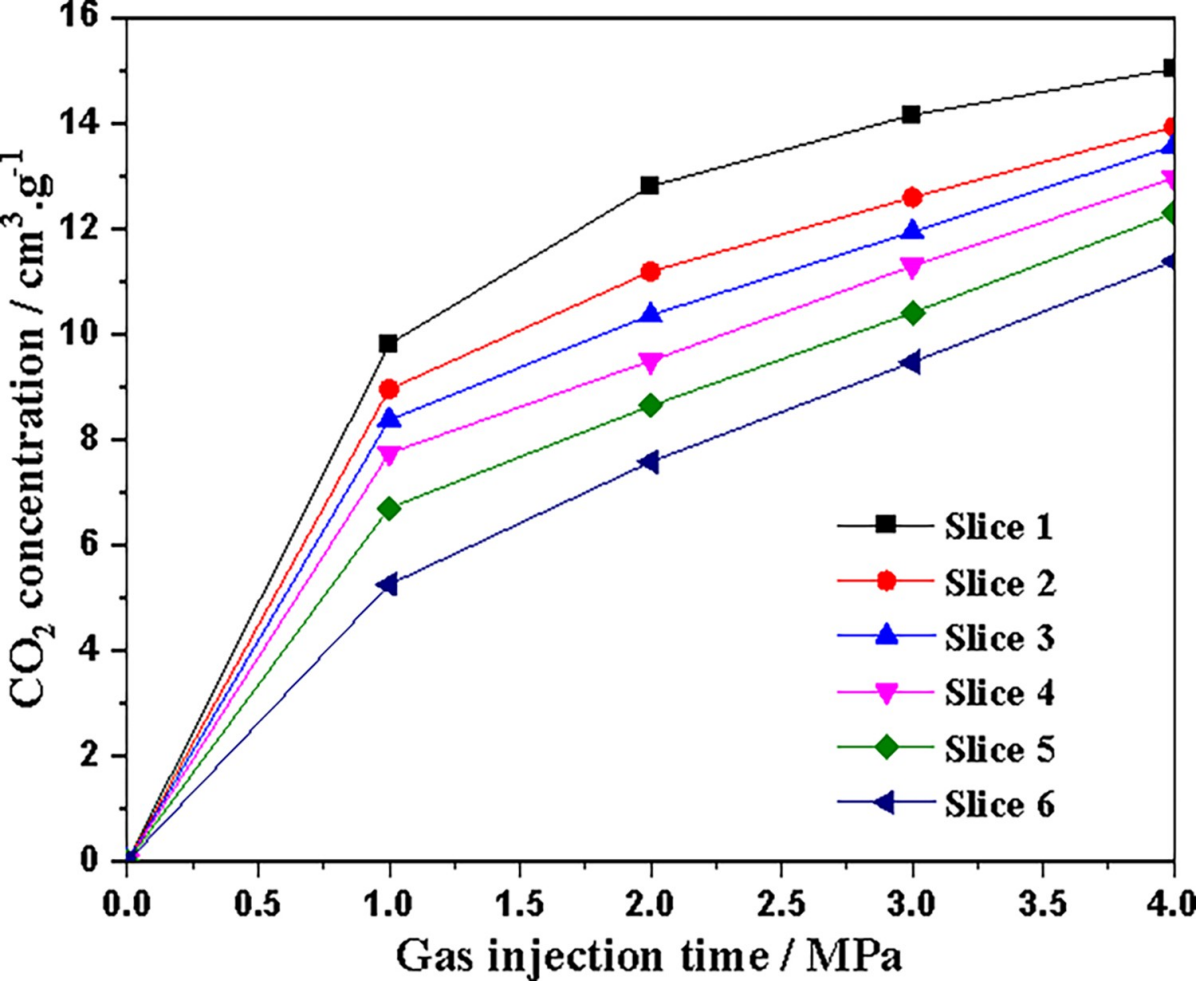
Apparent sorption isotherms for various slices.

Fig 10 shows the average CO_2_ concentration of coal core slices at different injection pressures (150 hours). CO_2_ concentration decreases almost monotonically along the coal core axis from the injection end to the outlet end. Fig 11 shows the apparent adsorption isotherms of different slices obtained from CT data. For slice 1 with the closest diffusion of CO_2_ injection, when the injection pressure is above 3.0 MPa, the CO_2_ adsorption capacity tends to stabilize approximately with increasing adsorption pressure, indicating that adsorption equilibrium may have been reached at the position of slice 1. At other locations, as the injection pressure increases, the CO_2_ concentration (adsorption capacity) also gradually increases, indicating that CO_2_ adsorption at this location has not yet reached equilibrium.

In summary, under constant temperature conditions, the influence of injection pressure is mainly reflected in two aspects:

1. Adsorption performance: Gas adsorption capacity in coal satisfies the Langmuir adsorption isotherm, which means that the higher the pressure, the greater the adsorption capacity of gas in coal. Therefore, in this experiment, it was observed that the CO_2_ concentration (adsorbed CO_2_) at coal core slice position was higher, as shown in Fig 8. The higher the injection pressure, the higher the CO_2_ concentration at the same coal core position with the same injection time (red represents CO_2_ concentration, and the higher the pressure, the greater the significance of red color in the same slice).
2. Gas permeability performance: The driving force for gas permeation in coal cores mainly comes from the gas pressure difference, and the larger the pressure difference, the faster the gas permeation. The higher the gas pressure at the injection end of coal core, the more significant the pressure difference formed in coal core, which can quickly drive gas to penetrate into the another end of coal core. In this process, the adsorption amount (CO_2_ concentration) is larger in coal core at the same slice location at the same time, the CO_2_ concentration in coal core slice in Fig 8 is more significant in red, and the CO_2_ concentration value in coal core in Fig 9 is larger. Moreover, under different injection pressures and with the same injection time, the CO_2_ concentration along the coal core axis shows a decreasing trend. Although this is a sign that gas in coal body has not reached adsorption saturation, the CO_2_ concentration value clearly indicates that when the injection pressure is higher, the CO_2_ adsorption amount is greater farther away from the injection end of coal core, which indicates that CO_2_ permeation in coal core is faster.

### 5.3 CO_2_ concentration in coal core after gas injection/desorption

The spatial distribution of CO_2_ density in the coal core after CO_2_ adsorption at 4.0MPa (injection pressure) and injection time of 150h is shown in Fig 6. The further away from the injection port the coal core slice contains less CO_2_, and there is no adsorption equilibrium at this time. In addition, it can be seen that the CO_2_ adsorbed at the coal core slices is uneven, and the upper left part of each slice adsorbs less CO_2_. This is because the upper left part of the coal core has more minerals and weaker CO_2_ adsorption ability.

**Fig 12:**
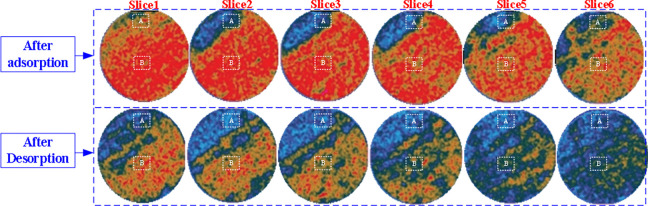
Changes of CO_2_ concentration in coal core after gas injection and desorption.

Taking the injection pressure of 4.0 MPa as an example, the spatial distributions of CO_2_ density in coal core are shown in Fig 12 after injection time of 150 hours and desorption time of 50 hours. From Fig 12, it can be seen that the coal core slices farther away from the injection inlet contain less CO_2_, and at these locations, they have not yet reached adsorption equilibrium. In addition, it can be seen that the CO_2_ adsorbed on the coal core slices is uneven, and the CO_2_ adsorbed on the upper left part of each slice is relatively small. This is because the upper left part of coal core has more minerals and weaker CO_2_ adsorption ability. After 50 hours of desorption, there is still a certain amount of CO_2_ in coal core, because during the desorption process, CO_2_ moves slowly in coal core and has not been completely desorbed. But overall, the closer to the exit end, the smaller the CO_2_ concentration and the more significant the decrease. From Fig 12, it can also be seen that the average CO_2_ concentration of the slice decreases almost monotonically along the coal core axis from the injection end to the outlet end. Meanwhile, CO_2_ concentration is uneven in each slice of coal core. CO_2_ concentration adsorbed is more significant in the lower right area of the slice, and less in the upper left area. This is because there are more minerals in the upper left area of the coal core, and its adsorption performance is weak.

In order to compare and analyze the non-uniformity of CO_2_ adsorption on coal core slices, two Regions A and B were selected on the coal core slices. Based on the CT values of Region A and B after adsorption and desorption, the difference in coal core density between these two areas can be calculated to obtain the CO_2_ adsorption concentration for each slice.

**Fig 13:**
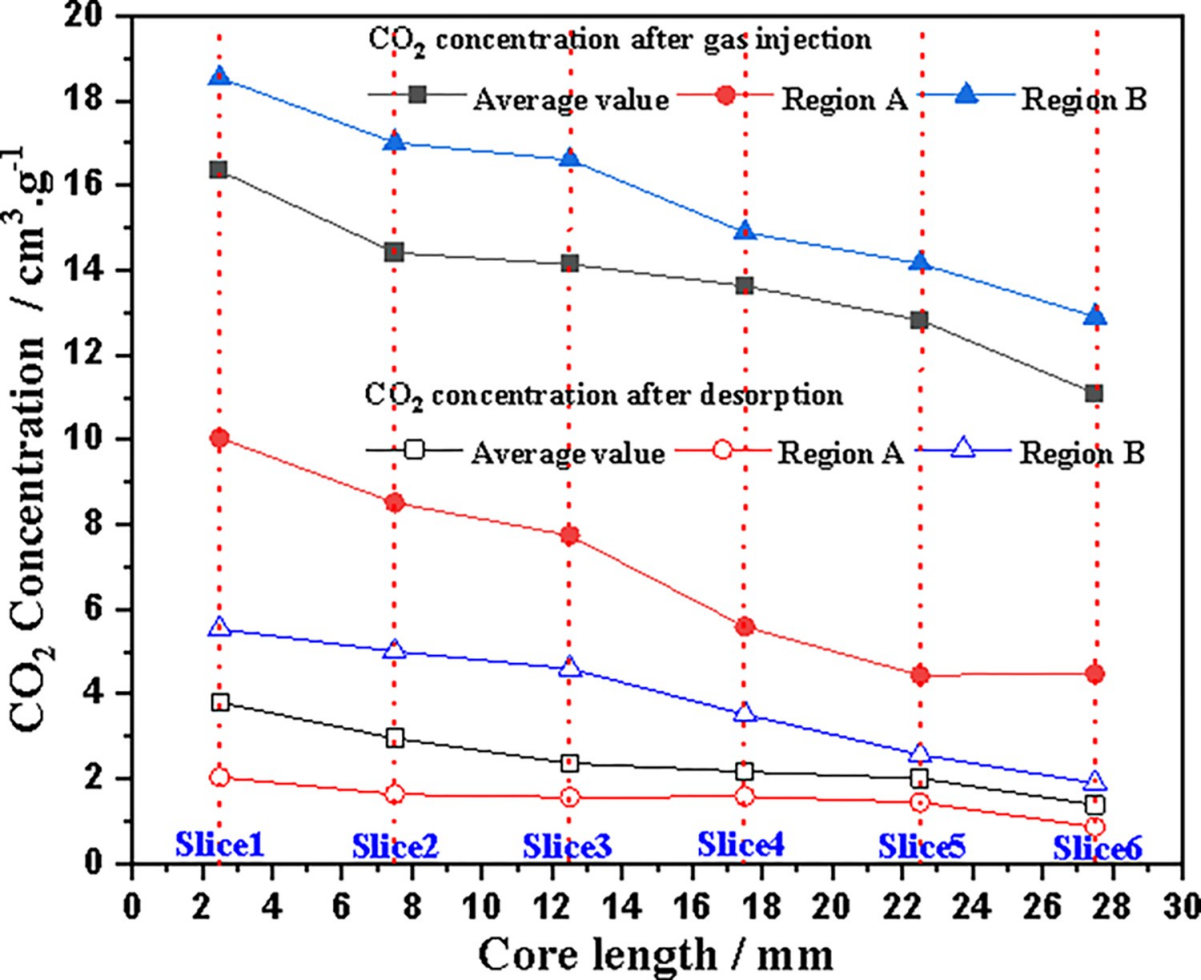
Relationship curves between CO_2_ concentration and coal core length for complete sections and two regions.

Fig 13 shows the axial variation of CO_2_ concentration in Regions A and B, as well as the entire slice, after 150 hours of gas injection (4.0 MPa) and 50 hours of desorption. These curves illustrate the non-uniformity of CO_2_ concentration in coal core. CO_2_ concentration in Region A is much lower than the average CO_2_ concentration in this slice, which also confirms the visual observation results in the pictures. However, CO_2_ concentration in Region B is much higher than that in Region A, usually higher than the average CO_2_ concentration. This is because there are more minerals in the upper left area of coal core, and its adsorption performance is weak. It was also observed that the higher the coal density at the slice, the lower the CO_2_ concentration. From Fig 13, it can also be seen that after 50 hours of desorption, the overall CO_2_ concentration in coal core decreases, and the average concentration approximately linearly decreases along the axial direction of coal core. For local areas, CO_2_ concentration in Region B decreases particularly significantly along the axial direction of coal core (with a large gradient), while CO_2_ concentration in Region A is lower, and CO_2_ concentration is smaller after desorption.

### 5.4 Characteristics of uneven adsorption of coal cores

**Fig 14:**
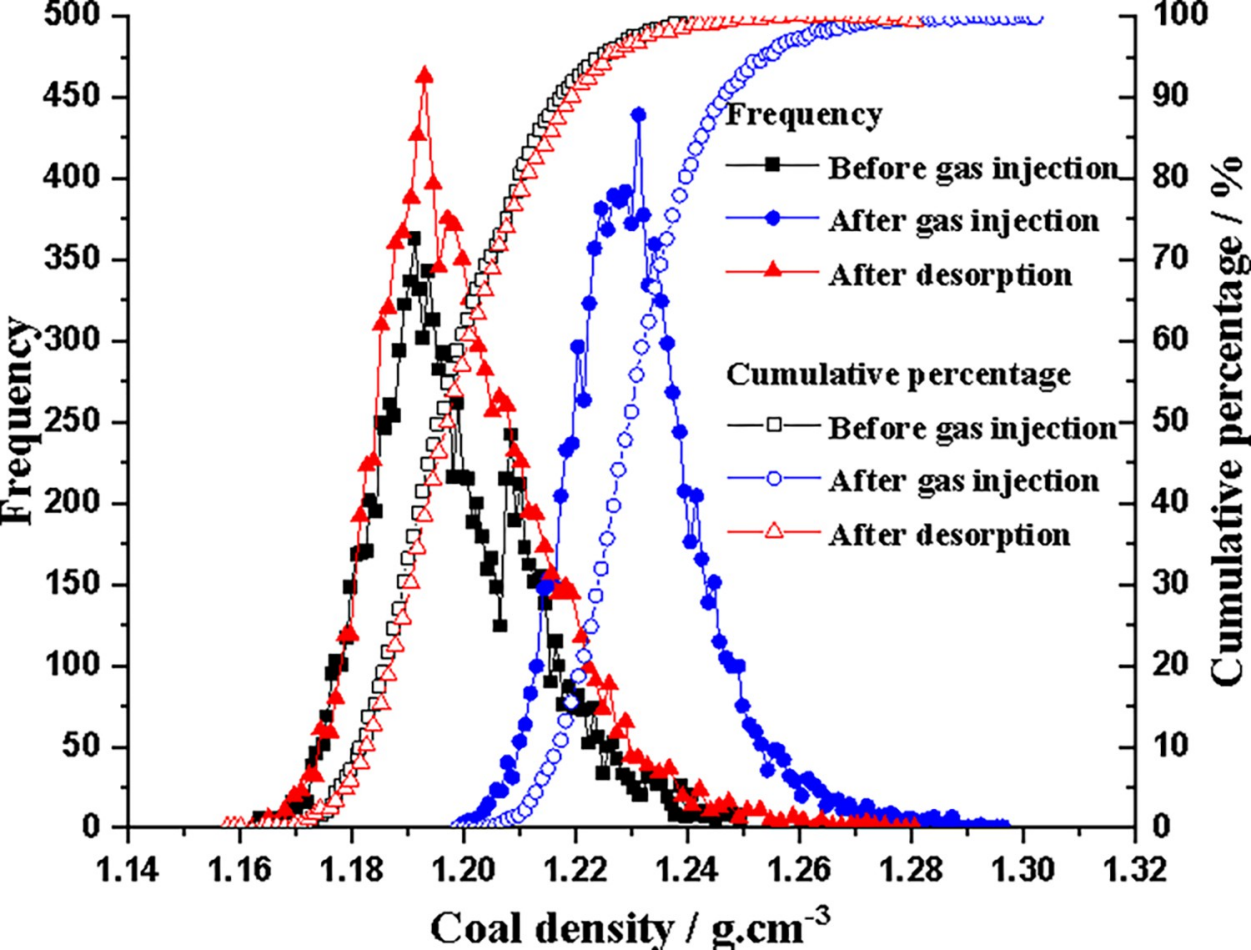
Voxel density of coal sample slice 1 before and after CO_2_ injection and desorption.

When a small region inside the coal core is selected for analysis, a very significant density change (non-uniformity) is observed compared to the average value of the coal core specimen. The calculated CO_2_ concentration in the high-density region of the coal core is significantly lower than the average CO_2_ adsorption amount in the slice. Here, a CT instrument was used to detect core heterogeneity at a single 3D pixel (voxel) scale (hexahedron of 0.25×0.25×2.0mm size per 3D pixel) with approximately 9900 voxels in each slice. Taking section 1 as an example, the frequency and cumulative percentage of coal core density at different voxels before gas injection, after gas injection and after desorption are shown in Fig 14.

In Fig 14, the voxel density of coal core slice 1 before gas injection is mainly concentrated between 1.16g/cm^3^ and 1.25g/cm^3^, with an average voxel density of 1.195g/cm^3^ and an average skewness factor (asymmetry measure) of 0.67 (completely symmetric distribution, skewness factor of 0). After gas injection, the voxel density of coal core slice 1 is mainly concentrated between 1.20g/cm^3^ and 1.30g/cm^3^, with an average voxel density increasing to 1.23g/cm^3^ and an average skewness factor of 0.88. After desorption, the voxel density of coal core slice 1 is mainly concentrated between 1.15g/cm^3^ and 1.28g/cm^3^, with an average voxel density of 1.199 mg/cm^3^, which is close to returning to the pre gas injection state (difference of 0.34%), and the average skewness factor of distribution is 0.82. Although the difference in coal voxel density before and after CO_2_ injection and desorption indicates that a certain amount of CO_2_ remains in the coal core, the frequency distribution and average voxel density of the coal core after desorption are close to that before gas injection.

**Fig 15:**
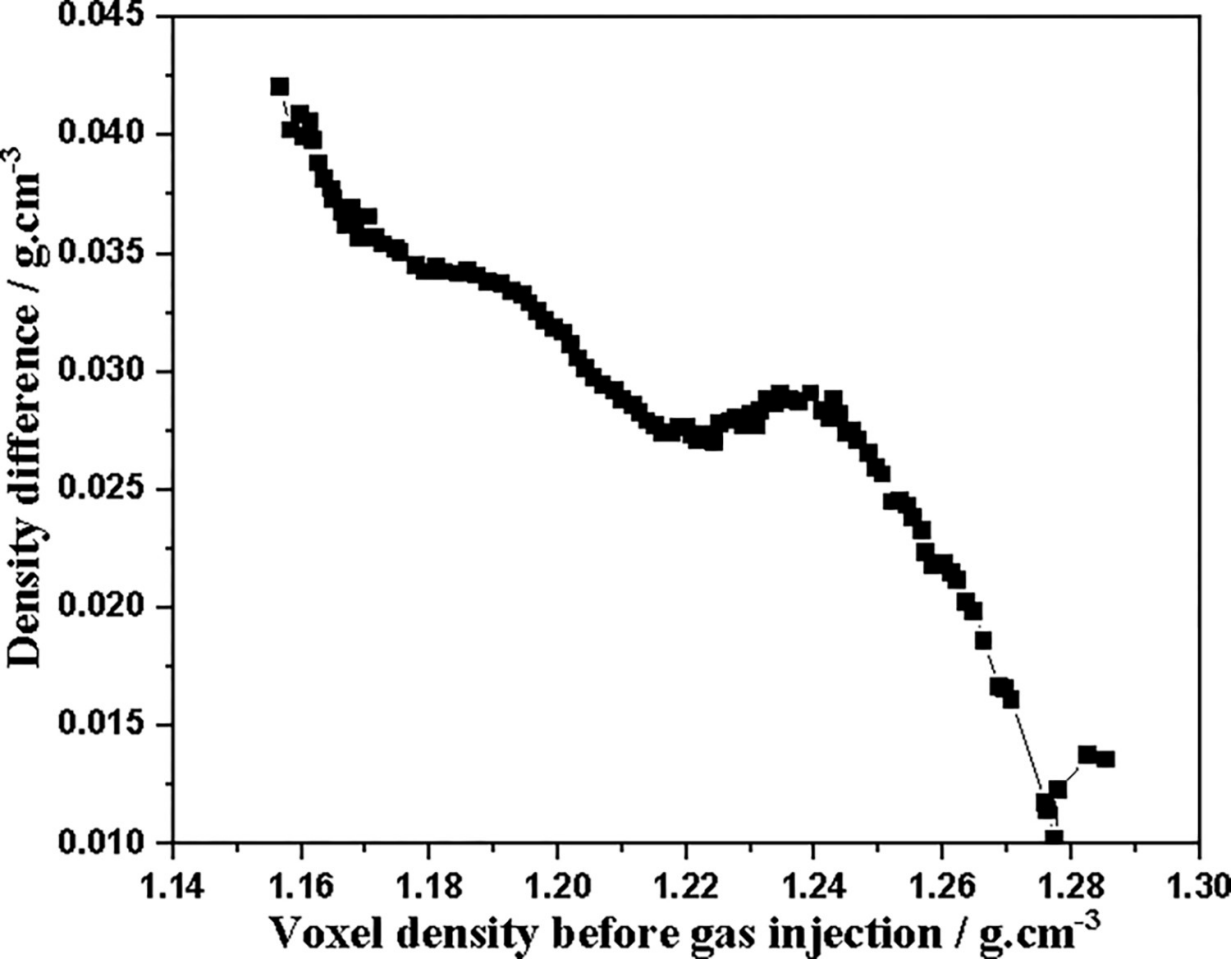
Increase in voxel density of slice 1 before and after CO_2_ injection into coal core.

At the same time, it can be seen from Fig 14 that the density of different voxels in slice 1 changes significantly, which also reflects the extremely uneven adsorption of CO_2_ by the coal core. The relationship between the voxel density difference caused by adsorption after CO_2_ injection (voxel density after adsorption minus initial voxel density) and the initial coal density is shown in Fig 15. Fig 15 shows that the low-density macerals (coal matrix) absorb more CO_2_ than the high-density macerals (minerals), which is also consistent with the results shown in Figs 12 and 13.

## 6 Conclusion

Using dual energy X-ray CT, the density change of coal core before and after gas injection was calculated, and the CO_2_ concentration in coal core was obtained. The influence of minerals in coal matrix on coal density was analyzed, and the characteristics of uneven adsorption of minerals on CO_2_ were revealed. The conclusions are as follows:

1. Due to the mineral composition in the coal core, the density distribution of the coal core has certain differences (uneven density). The coal core in the region where the mineral exists has higher density, while the density of the coal matrix is smaller. The error between the density of coal calculated by CT data and the density of coal core calculated by mass/volume method is less than 1.50%.
2. The average CO_2_ concentration of the slices increases with time, with higher CO_2_ concentration near the gas injection end and lower CO_2_ concentration far from the gas injection end. That is, CO_2_ concentration decreases almost monotonically along coal core axis from gas injection end to outlet end. Meanwhile, the higher the CO_2_ injection pressure, the higher the CO_2_ density at the same location within the same injection time.
3. The farther away from the injection inlet, the less CO_2_ the coal core slices contain, and the CO_2_ adsorbed by each slice of the coal core is also uneven. The higher the coal density (with more minerals) at the slice, the weaker the CO_2_ adsorption ability.
4. When the coal core is not fully desorbed (CO_2_ migrates slower in the coal core and takes longer), the closer it is to the outlet section, the smaller the CO_2_ concentration, and the more significant the decrease. The average CO_2_ concentration in the slice decreases approximately linearly along the axial direction of the coal core. For local regions, the higher the mineral content (higher coal density), the more significant the CO_2_ concentration changes along the axial direction of the coal core.
5. The average voxel densities of coal core slice 1 before and after gas injection and desorption are 1.195g/cm^3^, 1.23g/cm^3^, and 1.199 mg/cm^3^, respectively, and the average skewness factors of distribution are 0.67, 0.88, and 0.82, respectively. The significant changes in different voxel densities before and after gas injection indicate the extreme non-uniformity of CO_2_ adsorption by the coal core. There is little difference in the changes in different voxel densities before and after gas injection and desorption, indicating that the coal matrix recovers and approaches its original state after desorption.

## Supporting information

S1 Text Minerals in high density region of coal core. (XLSX)

S2 Text Liquid nitrogen and mercury intrusion test curves of coal samples (a) Liquid nitrogen test curve. Liquid nitrogen and mercury intrusion test curves of coal samples (b) Mercury intrusion test curve.
(ZIP)

S3 Text Specific surface area and pore volume of coal samples (a) Specific surface area. Specific surface area and pore volume of coal samples (b) Pore volume.
(ZIP)

S4 Text Relationship between CO_2_ concentration and injection time in different slices under different pressures (a) 1.0MPa. Relationship between CO_2_ concentration and injection time in different slices under different pressures (b) 2.0MPa. Relationship between CO_2_ concentration and injection time in different slices under different pressures (c) 3.0MPa. Relationship between CO_2_ concentration and injection time in different slices under different pressures (d) 4.0MPa.
(ZIP)

S5 Text Changes of CO_2_ concentration along the axial direction of coal core after gas injection. (XLSX)

S6 Text Apparent sorption isotherms for various slices. (XLSX)

S7 Text Relationship curves between CO_2_ concentration and coal core length for complete sections and two regions. (XLSX)

S8 Text Voxel density of coal sample slice 1 before and after CO_2_ injection and desorption. (XLSX)

S9 Text Increase in voxel density of slice 1 before and after CO_2_ injection into coal core. (XLSX)
